# Supplementary material for: The genome sequence of the Amphibious Bistort, Persicaria amphibia (L.) Delarbre (Caryophyllales: Polygonaceae)
Source: Wellcome Open Res. 2026 Oct 13;11:870. [Version 1] doi: 10.12688/wellcomeopenres.28121.1 (PMC13691359; doi:10.12688/wellcomeopenres.28121.1)
Supplement: Peer review history

# Reviewer report: Reviewer response for version 1

**DOI:** 10.21956/wellcomeopenres.30940.r175303

**Published:** 2026-10-22

**Authors:** Vojtech Zarsky

**Affiliations:** 1 University of Ostrava, Ostrava, Czech Republic

This is a genome note of the amphibious flowering plant, *Persicaria amphibia*. The genome was thoroughly sequenced using the long-read sequencing platform PacBio HiFi and illumina-based Hi-C method. This data was then used to generate assemblies of two haplotypes and the organellar genomes. Various metrics, including the high coverage, N50, k-mer-based and BUSCO-based completeness estimates indicate high-quality assemblies, with high contiguity and completeness. The haplotype 1 was resolved to 48 well-supported chromosomes. Comparison with other published genomes of the same genus suggests that this assembly conforms to a typical genome size and also to the size determined by flow cytometry. A poly(A)-selected RNA library was sequenced as a part of the project.

 The sample preparation, sequencing, assembly and data analysis have been conducted to the highest standards, resulting in a high-quality genome assembly. A logical next step will be generating gene annotations, which can be well aided by the RNA-seq data generated as part of this project. I would like to stress this, because as of now, there is no predicted proteome of any *Persicaria* representatives available (when checking Uniprot and NCBI) and I see this as a major gap for further genomics-based studies.

Are the datasets clearly presented in a usable and accessible format, and the assembly and annotation available in an appropriate subject-specific repository?

Yes

Are sufficient details of the sequencing and extraction, software used, and materials provided to allow replication by others?

Yes

Are the rationale for sequencing the genome and the species significance clearly described?

Yes

Are the protocols appropriate and is the work technically sound?

Yes

Reviewer Expertise:

Genomics, Algal research, Protistology, Organellar biology

I confirm that I have read this submission and believe that I have an appropriate level of expertise to confirm that it is of an acceptable scientific standard.

# Reviewer report: Reviewer response for version 1

**Published:** 2026-10-13

**Authors:** Automated Benchmarking

**Affiliations:** 1 Wellcome Sanger Institute, Hinxton, UK

As this article is a Genome Note, Automated Benchmarking has been used to determine whether the reported assembly passes four defined checks established by the Earth BioGenome Project: the raw and assembled data are publicly available, the Earth BioGenome Project three-part metric is met, the k-mer completeness exceeds 90%, and the BUSCO score passes the stated threshold. For Persicaria amphibia (dcPerAmph1), all four checks passed. Data available: pass. ENA study PRJEB76751, raw-read accessions ERR13304135, ERR13304137, ERR13304136, ERR13317819, and ERR15551459, and assembly accessions GCA_965605765.1 and GCA_965605725.1 are public. Earth BioGenome Project three-part metric: pass. Haplotype 1 is reported as 6.C.Q65, meeting the 6.C.Q40 reference standard. k-mer completeness: pass. The combined haplotype assemblies have 99.41% k-mer completeness, exceeding the Earth BioGenome Project target of 90%. BUSCO: pass. Haplotype 1, which is proposed as the reference assembly for this species, has 96.9% complete BUSCOs (6.7% single-copy and 90.2% duplicated). BUSCO software version: 6.0.0; lineage dataset: eudicots_odb10. It meets the plant-specific target of greater than 90% complete BUSCOs. This decision uses values reported in the Genome Note and public accession records.

Reviewer Expertise:

NA

I confirm that I have read this submission and believe that I have an appropriate level of expertise to confirm that it is of an acceptable scientific standard.
